# Titanium micro-particles are commonly found in soft tissues surrounding dental implants

**DOI:** 10.1038/s43856-025-00756-3

**Published:** 2025-03-18

**Authors:** Carlotta Dionigi, Gyula Nagy, Jan Derks, Yuki Ichioka, Cristiano Tomasi, Lena Larsson, Daniel Primetzhofer, Tord Berglundh

**Affiliations:** 1 Department of Periodontology, Institute of Odontology, Sahlgrenska Academy, University of Gothenburg, Gothenburg, Sweden; 2 Department of Physics and Astronomy, Uppsala University, Uppsala, Sweden; 3 Clinic of Periodontics, Public Dental Service, Region Västra Götaland, Gothenburg, Sweden; 4 Tandem Laboratory, Uppsala University, Uppsala, Sweden

**Keywords:** Dentistry, Gene expression analysis, Biophysical methods

## Abstract

**Background:**

Dental implants are one of the most frequently used medical devices for therapeutic purposes in dentistry. Peri-implantitis is a severe, microbial biofilm-associated condition, characterized by inflammation in peri-implant soft tissues and destruction of supporting bone. It has been suggested that metal particles originating from the implant may influence the local host response to microbial biofilms.

**Methods:**

Soft tissue biopsies were collected from implant sites with and without peri-implantitis in 21 patients. Micro Proton-induced X-ray Emission (µ-PIXE) analysis was used to localize, quantify and characterize titanium micro-particles within tissues. RNA sequencing was performed to evaluate potential associations between titanium micro-particles and gene expression profiles in peri-implantitis lesions.

**Results:**

Titanium micro-particles are consistent findings in soft tissues surrounding dental implants. Their occurrence varies across patients but not between sites with and without peri-implantitis within the same individual. Most particles reside in a 2-mm wide tissue portion close to the implant/tissue interface. The time in function of the implants does not influence the volumetric density of titanium micro-particles, while implant systems do. Fourteen differentially expressed genes are identified when comparing peri-implantitis samples with high and low densities of titanium micro-particles. The gene-set enrichment analysis reveals functions related to the regulation of the immune response and epithelial development.

**Conclusions:**

The present results indicate that titanium micro-particles are commonly found in tissues surrounding dental implants and are not associated with the occurrence of peri-implantitis.

## Plain language summary

Dental implants are frequently used in dentistry. Peri-implantitis is a severe condition in which micro-organisms such as bacteria grow excessively and cause inflammation in areas around the implants. This can result in destruction of supporting bone. It has been suggested that metal particles originating from the implant may influence how the body reacts to the presence of the bacteria. We analyzed samples of human soft tissue taken from implant sites with and without peri-implantitis. Titanium micro-particles were consistent findings in the soft tissue specimens regardless of the presence of peri-implantitis. The results indicate that titanium micro-particles are commonly found in tissues surrounding dental implants and are not associated with the occurrence of peri-implantitis.

## Abstract (web summary)

Dionigi et al. analyze human biopsy material collected from dental implant sites with and without peri-implantitis. Titanium micro-particles are found in soft tissues surrounding dental implants with the occurrence of titanium micro-particles varying across patients but not between sites with and without peri-implantitis.

## Background

Among the large number of medical devices available for therapeutic purposes, dental implants are one of the most frequently used. According to the European directives MDD, 93/42/EEC and the 2017 Medical Device Regulation (MDR, Council Regulation 2017/745), dental implants represent class IIb of medical devices, which also includes implants used in the orthopedic, ophthalmic, and cardiovascular fields. Dental implants are in this context defined as “non-active implants and long-term (>30 days) surgically invasive devices”^1^. While estimations from economic reports indicate that >20 million implants are installed annually worldwide, there are limited data on the proportion of individuals with dental implants from a population perspective. Results presented in a recent annual report from the Swedish Quality Registry on Caries and Periodontal Diseases (SKaPa)^2^ revealed that 4.7% of 3.3 million subjects >20 years had ≥1 dental implants. For subjects >70 years, the prevalence varied between 10% and 13% and has increased over the past 10 years. Thus, from a global perspective, subjects with dental implants constitute a large population.

Although the use of dental implants is considered a safe and predictable method to replace teeth, inflammatory conditions in peri-implant oral tissues occur. Peri-implantitis represents a severe, microbial biofilm-associated form of these conditions and is characterized by both inflammation in peri-implant soft tissues and destruction of supporting bone^3,4^. The disorder shares several critical features with periodontitis, its counterpart at natural teeth^5^. Cross-sectional studies on subjects with dental implants have shown that about 15% exhibit moderate/severe peri-implantitis^6–8^. The aggressive nature and rapid progression of peri-implantitis emphasize the need for accurate risk assessments, effective screening, and early detection^9^. Untreated peri-implantitis may lead to loss of implants, resulting in loss of function, impaired esthetics, and morbidity. Furthermore, treatment of peri-implantitis is, besides the inconvenience and discomfort for the patient, also demanding in terms of resources^10^.

Studies on human samples obtained from sites exhibiting peri-implantitis are characterized by large inflammatory cell infiltrates with high densities of plasma cells, B-cells, and neutrophils^11–14^. Dental implants are metal devices mainly comprised of titanium, and it has been suggested that micro-particles originating from the implant may influence and exacerbate the local host response to microbial biofilms residing on implants. Studies performed on small numbers of human samples from peri-implantitis sites have shown the occasional occurrence of titanium micro-particles in inflamed peri-implant tissues^15–19^. The significance of these reports, however, is unclear as no corresponding analyses were made of non-diseased tissues. Thus, an appraisal of the overall occurrence, size, and distribution of titanium micro-particles in healthy and diseased peri-implant tissues is required to unravel potential associations with peri-implantitis.

Here we report data from µ-PIXE analyses of human biopsy material. The purpose was to assess the occurrence and localization of titanium micro-particles in soft tissue specimens obtained from dental implant sites exhibiting either (i) severe inflammation together with evident destruction of supporting bone (peri-implantitis) or (ii) clinically healthy conditions or mild inflammation and no evident bone loss (reference sites). A further aim was to evaluate potential associations between concentrations of titanium micro-particles and gene expression profiles in peri-implantitis lesions. We demonstrate that titanium micro-particles are commonly found in tissues surrounding dental implants and are not associated with the occurrence of peri-implantitis.

## Methods

### Study population

Twenty-one patients with dental implant-supported crowns or prostheses [18 women/3 men; mean age 72 years old (SD 11 years; range: 45–90 years), 18 non-smokers/3 current smokers, 2 patients with type 2 diabetes] were consecutively recruited from the Specialist Clinic of Periodontics in Gothenburg, Public Dental Services, Region Västra Götaland, Sweden. The study protocol was approved by the Swedish Ethical Review Authority (Dnr 2021-00508). Before enrollment, all subjects received detailed information about the study protocol and signed an informed consent.

Each patient had ≥1 dental implants exhibiting (i) severe inflammation together with evident destruction of supporting bone (peri-implantitis) and (ii) ≥1 adjacent implants with clinically healthy conditions or mild inflammation without evident bone loss (reference sites). The clinical characteristics for peri-implantitis sites included peri-implant probing pocket depth (PPD) of ≥7 mm, bleeding and/or suppuration on probing (BoP/SoP) and radiographically assessed bone levels of ≥3 mm. Reference implant sites showed either presence or absence of BoP and PPD ≤ 5 mm. Information on implant characteristics, i.e., implant system, material, surface topography, and time in function, was noted (Table 1).

**Table 1:** Implant characteristics and clinical measurements

	Peri-implantitis			Reference implant		
	*n*	*mean / %*	*sd*	*n*	*mean / %*	*sd*
Implant characteristics						
Years in function	18	12.6	5.46	18	12.6	5.46
Diameter (mm)	18	4	0.7	18	3.9	0.7
Length (mm)	18	12.7	3.1	18	13.2	2.8
Jaw						
Mandible	6	33.30%		6	33.30%	
Maxilla	12	66.70%		12	66.70%	
Location						
Anterior	5	27.80%		7	38.90%	
Posterior	13	72.20%		11	61.10%	
Implant System/Surface						
Ankylos/Friadent Plus (Dentsply Sirona)	1	5.60%		1	5.60%	
Astra Tech/Osseospeed (Dentsply Sirona)	5	27.80%		5	27.80%	
Astra Tech/TioBlast (Densply Sirona)	2	11.10%		2	11.10%	
Brånemark/Turned (Nobel Biocare)	2	11.10%		2	11.10%	
Nobel/TiUnite (Nobel Biocare)	7	38.90%		7	38.90%	
Straumann/SLA (Straumann)	1	5.60%		1	5.60%	
Tooth Reconstruction Type						
Single crown	3	16.70%		3	16.70%	
Two-unit bridge	6	33.30%		6	33.30%	
Three-unit bridge	1	5.60%		1	5.60%	
Multiple-unit bridge	2	11.10%		2	11.10%	
Full-arch bridge	3	16.70%		3	16.70%	
Overdenture	3	16.70%		3	16.70%	
Tooth Reconstruction Retention						
Cemented	1	5.60%		1	5.60%	
Screw-retained	17	94.40%		17	94.40%	
Clinical measurements						
Probing pocket depth (mm)	18	7.6	0.78	18	4.1	1.08
Radiographic bone level (mm)	18	4.7	0.96	18	1.6	0.77
Bleeding on probing (%)	18	100%		18	66.70%	
Suppuration on probing (%)	18	38.90%		18	0%	

Patients were excluded if the target implant with peri-implantitis and the reference implant differed in terms of time of installation and/or implant system, or if target implants had already undergone a surgical intervention for peri-implantitis. Additional exclusion criteria were the use of systemic/local antibiotics over the last 6 months and presence of systemic conditions affecting peri-implant tissues and/or impeding the surgical intervention (e.g., uncontrolled diabetes, immunosuppressive medication). Upon oral hygiene instructions and professional supra-gingival cleaning, patients were scheduled for surgical treatment, which included collection of the soft tissue biopsies from peri-implantitis and adjacent reference implant sites.

### Surgical intervention and biopsy retrieval

**Fig. 1:**
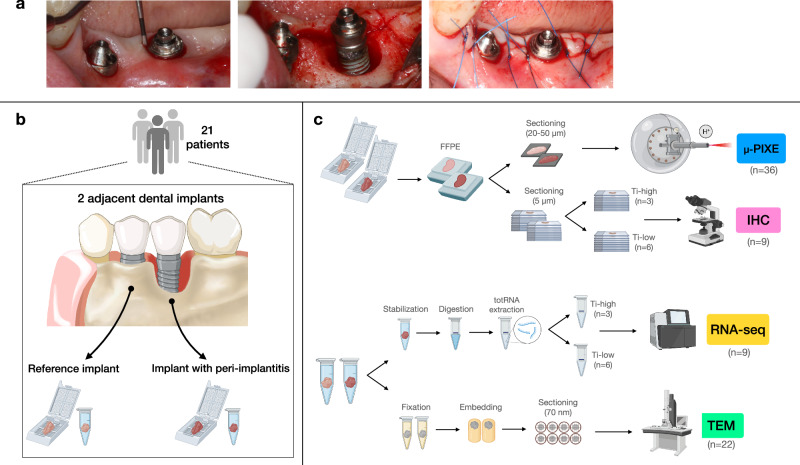
Study outline. Illustrations created with BioRender. **a** Clinical pictures from one representative patient. Reference implant (left) and implant with peri-implantitis (right) depicted before, during, and after the surgical intervention. **b** Schematic illustration of soft tissue biopsy retrieval. Twenty-one patients were recruited. One soft tissue biopsy, about 3–5 mm wide, was collected from one dental implant site with peri-implantitis and from one adjacent reference implant site. An additional tissue portion, about 1 mm wide, was collected from the same peri-implant sites in each patient. **c** Schematic illustration of soft tissue biopsy processing and applied methods for analysis. - Formalin-fixed paraffin-embedded samples were prepared for the localization, quantification, and characterization of titanium micro-particles. Three pairs of specimens (*n* = 6) were excluded from the analysis due to complications in sample preparation. Thus, a total of 36 sections, 20–50 μm thick, were analyzed with Micro Proton-Induced X-ray Emission (μ-PIXE). Three additional sections obtained from patients affected by periodontitis (i.e., with no dental implants) were used as negative controls. - Peri-implantitis specimens obtained from 10 patients were prepared for bulk RNA-sequencing analysis (RNA-seq). Specimens were divided in two groups depending on the volumetric density of titanium particles found in the corresponding FFPE samples. Differential gene expression analysis was performed comparing samples presenting with volumetric densities higher than the mean (Ti-high) to those presenting volumetric densities lower than the mean (Ti-low). One sample was excluded from the analysis due to the exclusion of the corresponding FFPE specimen. Thus, a total of 9 samples were analyzed. - Sections from 9 peri-implantitis FFPE (matched to samples analyzed with RNA-seq) were prepared for immunohistochemistry (IHC). - Samples from peri-implantitis and reference implant sites obtained from 11 patients were analyzed with transmission electron microscopy (TEM).

When feasible, implant-supported reconstructions were disconnected prior to the surgical intervention. Following local anesthesia, a crestal incision was made with a stainless-steel blade (no 15c; Swann-Morton, Sheffield, England) and, prior to flap elevation, a soft tissue biopsy, about 3–5 mm wide and extending from the soft tissue margin to the bone crest, was carefully dissected from both the diseased and the adjacent reference implant sites using universal stainless-steel curved tweezers (5–107, Bent/Finely serrated, 150 mm, Bernstein) (Fig. 1a). Biopsies were rinsed with saline, mounted in plastic cassettes (Tissue-Tek Paraform Sectionable Cassette System; Sakura Finetek Europe, Netherlands) and placed in 4% buffered formalin for 48 h. The tissue samples (named “FFPE samples”) were stored in 70% ethanol, kept at 4 °C, subsequently dehydrated and embedded in paraffin until further processing for µPIXE and IHC analyses (Fig. 1c). After flap elevation, a 1-mm wide peri-implant connective tissue portion was dissected at the same “FFPE sample” collection sites, both at peri-implantitis and reference implants (Fig.1b). All tissue samples were rinsed with saline and immediately placed in Eppendorf tubes according to the following outline: (i) samples from the initial 11 patients (named “TEM samples”) were immersed in Karnovsky fixative, kept at 4 °C for a maximum of 14 days and prepared for transmission electron microscopy analysis; (ii) samples from the remaining 10 patients (named “RNA-seq samples”) were immersed in RNAlater (AMBION, Inc., Austin, Texas, USA), kept at 4 °C for 48 h and stored at −80 °C until further processing for RNA-sequencing.

At peri-implantitis sites, remaining inflamed tissues were removed and the implant surface was mechanically decontaminated using titanium curettes and a rotating titanium brush (Nano NiTi Brush, HANS KOREA CO. Ltd) under continuous irrigation with saline. Flaps were replaced and sutured (Fig. 1a) and implant-supported prostheses were reconnected.

### Micro Proton-induced X-ray Emission (µ-PIXE)

#### Elemental maps acquisition

From each FFPE sample (*n* = 42), a single 20/50-µm-thick section was obtained with a microtome equipped with a stainless-steel knife, individually mounted on 1 × 1 cm silico-crystal substrates and dewaxed at 60 °C overnight. All sections remained uncoated, were first imaged under an inverted light microscope (Leica, Wetzlar, Germany) at 5× magnification and then transferred to the Tandem Laboratory (Uppsala University, Sweden). Samples from three patients had to be excluded due to complications during sample preparation. Thus, a total of 18 paired FFPE-samples were included in the final analysis (*n* = 36). For control purposes, tissue samples from periodontitis sites in 3 patients were also prepared.

The samples were characterized by µ-PIXE analysis at the scanning nuclear microprobe of the Tandem Laboratory of Uppsala University. A beam of 2 MeV H^+^ ions was used for the measurements, focused to 4–5 micrometers in diameter. The scanning system of the microprobe was modified to combine beam- and stage-scanning in a “mosaic-scan” way^20^. Thus, large samples (up to several cm^2^ in size) could be mapped in detail in a time-efficient manner. Each specimen was divided into a number of sub-regions, over which mapping was performed by the beam-scanning method using a 200 nC/mm^2^ proton fluence. After the scan of each sub-region, individual titanium micro-particles were located in the raw titanium (Ti) µ-PIXE maps using a Hough-transformation-based object-identification algorithm. After identification of the particles, high statistics μ-PIXE measurements (20,000 nC/mm^2^ proton fluence) were performed in-situ on selected particles. Finally, the stage-scan method moved the sample to the next sub-region, where the whole procedure restarted until the entire specimen was scanned by the proton microbeam.

The acquired μ-PIXE spectra were analyzed using the GeoPIXE software (version 8.6, CSIRO, Australia) in order to generate true elemental maps^21^. Lower concentration thresholds were obtained by the use of false detections of the particle-searching algorithm, in which the high statistics μ-PIXE measurements revealed the absence of Ti. These thresholds were then used to filter out noise while keeping all the micro-particles, resulting in clean, background-free Ti maps suitable for image analysis.

A total of 39 samples (18 paired peri-implant tissue samples and 3 additional, negative control, periodontitis samples), altogether about 400 mm^2^, were fully mapped. Silico-crystal substrates were the source of Si-peaks in recorded spectra.

#### Image analysis

The elemental composite images, resulting from concentration maps obtained by the GeoPIXE software, were analyzed with the computerized image analysis software Image-Pro Premier (IPP, version 10, Media Cybernetics Inc., Rockville, MD, USA) by one trained investigator (C.D.). Sulfur maps were used to depict the entire outline of each specimen with a mouse cursor and compared, by superimposition, with the images acquired with the inverted light microscope (5× magnification). The entire outline of the specimen was then transferred to its paired titanium composite map. Paired sulfur and titanium composite maps were captured with identical size and a fixed 72 ppi resolution, thus allowing for an optimal transfer of data from one another. Image calibration was performed manually on each composite image using the side of a single tile as the reference line (=1 mm long).

The “smart segmentation tool” of the IPP software was used to generate an algorithm (accounting for color, intensity, morphology, and size/shape of particles) that was used for the identification of each particle. Different regions of interest (ROI) were depicted in each sample: the “entire specimen” corresponded to the entire biopsy, “zone 1” extended from the implant/tissue interface up to 1 mm, “zone 2” from 1 to 2 mm and “zone 3” from 2 mm and onwards. Additionally, one “zone with inflammation” and one “zone without inflammation” were determined in each specimen by superimposing the titanium maps over images obtained from 5-µm-thick Hematoxylin/Eosin-stained sections.

In each ROI, the total area (mm^2^) and volume (mm^3^) (=area of ROI × biopsy thickness), the number of titanium micro-particles (n) and the portion of the ROI area occupied by titanium micro-particles (%) were measured. The volumetric densities of titanium micro-particles were mathematically computed and expressed as number of micro-particles/mm^3^. For single-particle characterization, data on the micro-particle area, diameter, Feret diameter, and circularity were also collected. Vertical distances (mm) from the mucosal margin and horizontal distances (mm) from the implant/tissue interface were calculated for each particle. After tracing two reference lines corresponding to the implant/tissue interface and the mucosal margin, perpendicular (=minimum) distances were automatically measured for each particle using the “relative minimum distance between objects” measurement tool of the IPP software. The maximum width (mm) and depth (mm) of the specimens were also recorded.

### RNA-sequencing

#### Homogenization and Total RNA extraction

The “RNA-seq” samples obtained from peri-implantitis sites in 10 patients were immersed in RNAlater (AMBION, Inc., Austin, Texas, USA), kept at 4 °C for 48 h and stored at −80 °C. The RNeasy Plus Micro Kit (Quiagen) was used for the extraction of total RNA following the manufacturer’s instructions. In brief, samples were lysed and homogenized in a highly denaturing guanidine-isothiocyanate-containing buffer using TissueRuptor II, to ensure intact isolation of RNA content. The homogenized lysate was centrifugated for 3 min at maximum speed. After removal of supernatant by pipetting and transfer to a gDNA Eliminator spin column, a new centrifugation cycle in the column, in combination with an optimized high-salter buffer, allowed for the efficient removal of any genomic DNA. Ethanol was added, and the samples were applied to the RNeasy spin column to remove contaminants. The total RNA content was then eluted in 14 µl of RNAse-free water and stored at −80 °C. The integrity and size distribution of total RNA was checked with the Agilent Tapestation 4200 system. Quality check revealed that all samples presented with optimized concentrations of total RNA [range 34–463 ng/μL] and RIN scores [range 5–9]. One sample was excluded from the analysis as the density of titanium particles in the corresponding FFPE sample could not be assessed. Samples were divided into “Ti-low” (*n* = 6) and “Ti-high” (*n* = 3) groups depending on volumetric densities of Ti micro-particles being lower or higher than the mean (Supplementary Fig. 2).

#### Library preparation and analysis

Library preparation was conducted at the CORE Facility—Genomics (Sahlgrenska Academy, University of Gothenburg, Sweden) following the Illumina Stranded mRNA Prep Ligation protocol. In brief, oligo(dT) magnetic beads were used to capture mRNAs with polyA tails. The RNA was then fragmented and primed for first strand complementary DNA (cDNA) synthesis. The hexamer-primed RNA fragments were reverse-transcribed including Actinomycin D, granting RNA-dependent synthesis and improved strand specificity while preventing spurious DNA-dependent synthesis. The RNA template was then removed, and a replacement strand was synthesized to generate blunt-ended, double-stranded cDNA fragments. Deoxyuridine triphosphate was incorporated, and an adenine (A) nucleotide was added to the 3ʹ ends of the blunt fragments. A corresponding thymine (T) nucleotide on the 3ʹ end of the adapter was used for ligating the adapter to the fragment. Pre-index anchors were ligated to the ends of the double-stranded cDNA fragments to prepare them for dual indexing. After 11 PCR cycles and purification of the adapter-ligated fragments with magnetic beads, the libraries were normalized down to 1 nM, pooled together, diluted to 0.5 nM and standard run on a S2 flowcell on the NovaSeq 6000 (Illumina).

Data analysis was performed for differential gene expression and gene ontology in collaboration with the CORE Facility - Bioinformatics (Sahlgrenska Academy, University of Gothenburg, Sweden). The quality of the reads was examined using fastqc/0.11.9^22^, the resulting quality reports were summarized using MultiQC/1.9^23^. The reads were quality filtered using Trim Galore/0.4.0^24^ and adapters were removed using Cutadapt/1.9^25^. The quality filtered reads were aligned towards the human reference genome GRCh38.109 using STAR/2.7.10b^26^. Infer experiment within RSeQC/5.0.1^27^ was used to extract the strandness of the data. Featurecounts within the subread/2.0.4 package^28^ was used to gather the gene counts. The differential expression analysis was run in the R/4.1.3 package^29^ DESeq2/1.34.0^30^. Genes were considered differentially expressed with adjusted *p *< 0.05. Log-fold change was used to identify the magnitude of change in gene expression between groups. Threshold levels of Log_2_FC ≥ 1 and Log_2_FC ≤ -1 were applied to identify the most significantly differentially expressed genes. The package pheatmap/1.0.12^31^ was used to generate the heatmaps. ClusterProfiler/4.2.2^32^ was used to perform the overrepresentation analysis for Gene Ontology^33^ and Reactome^34^.

### Immunohistochemistry

From 9 “FFPE samples” (matching the 9 RNA-seq samples), 5-μm-thick serial sections were produced using a microtome, dewaxed, and incubated in DIVA antigen-retrieval solution (Biocare Medical, Histolab, Concord, CA, USA) at 60°C overnight. Incubation with 3% bovine serum albumin (BSA) in phosphate-buffered saline was performed to block unspecific binding. Primary antibodies were incubated for 30 min followed by incubation with Envision horseradish peroxidase-labeled polymer (Agilent, Santa Clara, CA, USA) for 30 min. Positive cells were detected using DAB substrate (Agilent). The choice of antibodies used for the immunohistochemical preparations were selected from data obtained by the RNA-seq analysis. Details on the dilutions and preparations used for the selected markers are presented in Supplementary Table 2. Counterstaining was performed with Hematoxylin. Sections were mounted, and cover slipped. Human oral mucosa tissue sections were used as positive controls, while negative controls were produced by substituting the primary antibody with non-immune serum.

Each section (total *n* = 45) was captured with a Glissando Desktop Scanner (Objective Imaging Inc, Kansasville, WI, USA) with a 20× magnification and transferred to a computer equipped with the computerized image analysis software Image-Pro Premier (IPP, version 10, Media Cybernetics Inc., Rockville, MD, USA). The infiltrated connective tissue (ICT) lateral of the pocket epithelium was depicted as ROI. For each marker, a differential segmentation analysis of color, intensity, morphology, and size was used to identify positive cells. Thus, the total area (mm^2^) of the ICT, the total number of positive cells (*n*) and the relative area occupied by positive cells (%) were measured. Cellular densities were mathematically computed and expressed as number of positive cells/mm^2^.

### Transmission electron microscopy

The “TEM samples” (*n* = 22) were profusely washed with saline and individually immersed in Eppendorf tubes with Karnovsky fixative and transferred to the Core Facility—Cellular Imaging (Sahlgrenska Academy, University of Gothenburg, Sweden) for further processing. All samples were moved into new tubes containing 1:10 diluted Karnovsky fixative with cacodylate buffer 0.1 M at 4 °C for a maximum of 14 days. Post-fixation was performed with Osmium tetra-Oxide (OsO4) 1% (EMS, US) plus Potassium Ferrocyanide 1% for 45 min in 0.05 M cacodilate buffer at room temperature. The Leica EM AMW Automatic Microwave Tissue Processor (Leica, Austria) was used for the following steps: Thio-Carbo-Hydrazide 1% (EMS, USA) for 10 min in water and OsO4 1% in water at RT for 20 min at 37 °C (O-T-O protocol). After profuse washing cycles with distilled water the samples were incubated with Uranyl Acetate 1% for 30 min at RT. The samples were dehydrated stepwise, with increasing concentrations of ethanol (Fisher Scientific, UK), switched to Propilene Oxide (Fisher Scientific, UK), and increasing concentrations of Hard-Plus epoxy resin (EMS, USA) without accelerator. Finally, the samples were washed several times with distilled water and embedded in 100% Hard-Plus epoxy resin with accelerator before polymerization at 60 °C for 16 h.

The polymerized specimens were trimmed with a 45° trimming diamond knife (Diatome, Switzerland) with a clearance angle of 6° into a pyramid shape to obtain a rectangular flat cutting surface. Alternate 70-nm-thick sections were produced using the UC6 ultramicrotome (Leica, Austria) equipped with a 45° diamond knife and clearance angle of 6°. The cutting was performed with a speed of 0.8 mm/s. The sections were collected onto 150 mesh copper formvar/carbon-coated grids (Agar Scientific Ltd, UK).

Grids were imaged using the Talos L120C transmission electron microscope (Thermofisher Scientific, USA) operating at 120 eKv. Micrographs for qualitative evaluations of metallic-like deposits were acquired with a CMOS 4K×4K Camera (Gatan, UK) using the TIA Software (Zeiss, Germany) at various magnifications (range: 1600×–28,000×).

### Statistics and reproducibility

The study’s design allowed for intra-individual comparison between peri-implantitis and reference sites. Mean values, standard deviations and confidence intervals were calculated for each variable. Due to a non-normal distribution of the data, median values and inter-quartile ranges were also calculated. Wilcoxon pairwise signed-rank test was used for comparisons between peri-implantitis and reference implant sites. A linear regression analysis was performed using the volumetric density of titanium micro-particles as the dependent variable, while implant characteristics (implant system, years in function, implant length, implant diameter, jaw, location, tooth-reconstruction type) and clinical measurements (probing pocket depth, marginal bone level) were used as independent parameters. The Mann-Whitney U test was used for comparisons of densities of positive cells between Ti-high and Ti-low groups. Statistical tests were performed using the Stata software 17.0 (StataCorp, USA). Differences between groups were considered significant with *p* < 0.05.

### Reporting summary

Further information on research design is available in the Nature Portfolio Reporting Summary linked to this article.

## Results

### Micro Proton-induced X-ray Emission

We demonstrated that titanium (Ti) micro-particles were consistent findings in soft tissues surrounding dental implants. While volumetric densities of titanium micro-particles varied markedly between patients, intra-individual comparisons revealed almost similar concentrations in specimens representing either severe inflammation and peri-implant bone loss or mild inflammation and no bone loss. We collected soft tissue samples from patients presenting with ≥1 dental implants with peri-implantitis and ≥1 reference implants (Fig. 1). The intra-individual design of the present study is unique and allows for detailed appraisal of potential associations between titanium micro-particles and peri-implantitis lesions. Thus, target and reference implants in each respective patient presented with identical characteristics regarding implant system, material, surface topography, installation procedure, and time in function (Table 1).

**Fig. 2:**
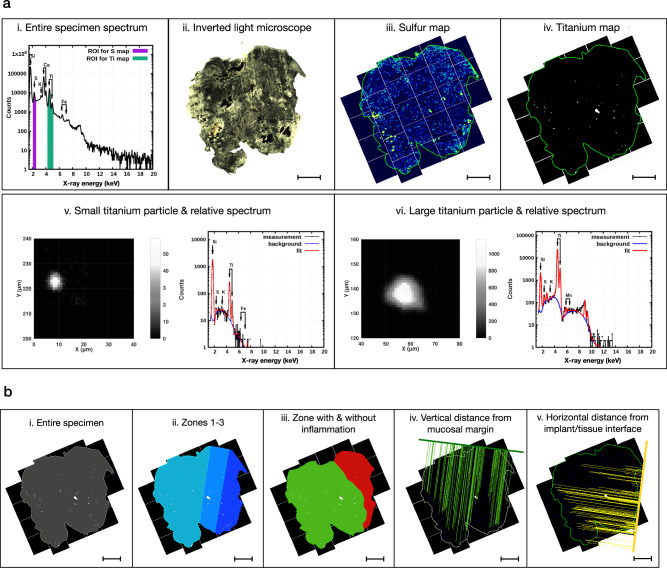
Micro Proton-induced X-ray Emission (μ-PIXE) - Image acquisition and analysis. The implant/tissue interface is found on the right side of the sample in all images. **a** (**i**): Representative μ-PIXE spectrum relative to the entire soft tissue specimen, highlighting the regions of interest (ROIs) for map definition: sulfur (S) in purple, titanium (Ti) in green. **a** (**ii**): Representative image of a soft tissue specimen obtained by inverted light microscopy (ILM). Magnification: 5×. Scale bar: 1 mm. **a** (**iii**): Representative μ-PIXE map (S) of the entire soft tissue specimen. The sulfur map is compared with the ILM image and used to outline the perimeter of the entire specimen (green line). Scale bar: 1 mm. **a** (**iv**): Representative μ-PIXE map (Ti) of the entire soft tissue specimen. The perimeter of the specimen (green line) is obtained from the sulfur map. Scale bar: 1 mm. **a** (**v**): Characterization of one representative small (diameter ≤ 15 μm) titanium micro-particle and its relative μ-PIXE spectrum. **a** (**vi**): Characterization of one representative large (diameter > 15 μm) titanium micro-particle and its relative μ-PIXE spectrum. **b** (**i**): Depiction of the “entire specimen” ROI (in gray) used for image analysis. **b** (**ii**): Depiction of the “zone 1” (in dark blue—from the implant/tissue interface up to 1 mm), “zone 2” (in blue—from 1 to 2 mm) and “zone 3” (in light blue—from 2 mm and onwards) ROIs used for image analysis. Scale bar: 1 mm. **b** (**iii**): Depiction of the “zone with inflammation” (in red) and “zone without inflammation” (in green) ROIs used for image analysis. Scale bar: 1 mm. These two ROIs were determined by superimposing the Ti-maps over the corresponding Hematoxylin/Eosin-stained tissue sections prepared for immunohistochemical analysis (see Fig. 4a). **b** (**iv**): Illustration of the vertical linear measurements. The vertical distances between the mucosal margin and all the Ti micro-particles found in the tissue section are measured using the “minimal distance tool” of the image analysis software. This feature automatically draws perpendicular lines from the center of each particle to the reference line representing the mucosal margin (thick green line). Scale bar: 1 mm. **b** (**v**): Illustration of the horizontal linear measurements. The horizontal distances between the implant/tissue interface (thick yellow line) and all the Ti micro-particles found in the tissue section are measured using the “minimal distance tool” of the image analysis software. Scale bar: 1 mm.

We applied μ-PIXE analysis for element-sensitive mapping with μm-resolution over 4–6 mm wide soft tissue samples. An example of a PIXE-spectrum integrated over the whole specimen and illustrating the element-specific signals is depicted in Fig. 2a (i). To ensure an efficient characterization of micro-particles, a new system for measurements, detailed in the Methods section, was introduced. Sulfur was detected ubiquitously within the tissue specimens and was, hence, used together with inverted light microscope images for orientation and mapping of tissue samples (Fig. 2a; ii–iii). During the mapping process, titanium micro-particles were detected and single-particle maps were acquired from selected micro-particles to further characterize the objects. Background subtraction was performed on the full-sample maps, resulting in clean, background-free elemental maps (Fig. 2a; iv–vi). Metal micro-particles with a diameter ≥1.5 µm dispersed in an organic matrix have been shown to be efficiently detected using µ-PIXE at beam conditions comparable or identical to the present investigation^35^.

**Fig. 3:**
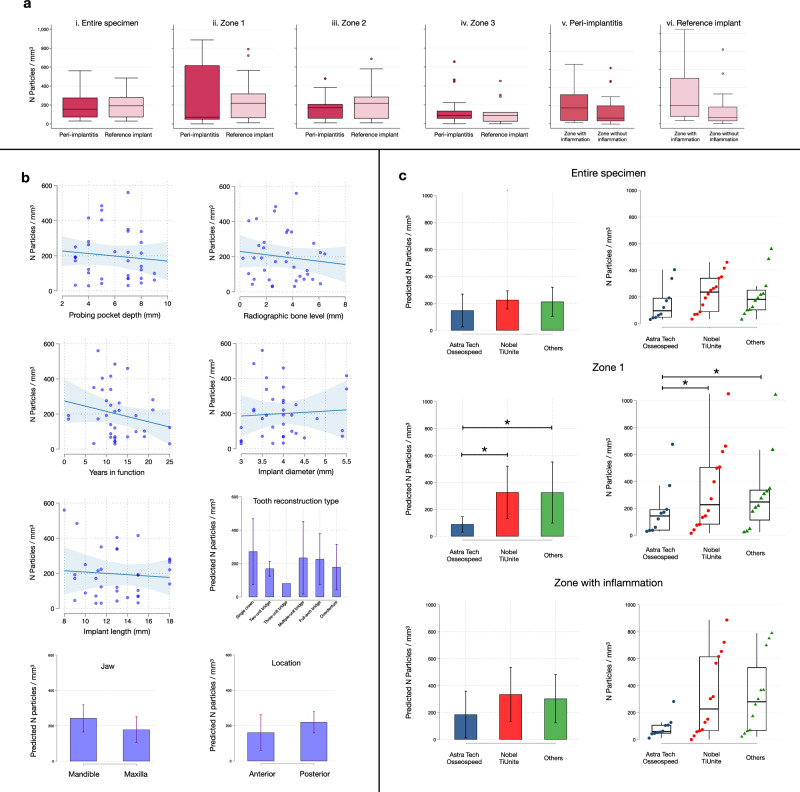
Results of μ-PIXE analysis. **a** Boxplots illustrate the volumetric density of titanium micro-particles observed in different ROIs in peri-implantitis (*n* = 18) and reference implant samples (*n* = 18; with exception of Zone 3 where *n* = 15). Median and IQR. Circles represent outliers. **b** Results of linear regression analysis: clinical measurements and implant-related characteristics. Scatterplots: each dot represents one implant (*n* = 36); bar graphs: mean and 95%CI. **c** Results of linear regression analysis: volumetric density of titanium micro-particles by implant system observed in the “entire specimen”, “zone 1” and “zone with inflammation” ROIs. Implant systems were grouped in three categories: Astra Tech Osseospeed (*n* = 10), Nobel TiUnite (*n* = 14), Others (*n* = 12). More details can be found in Table 1. Bar graphs: mean and 95% CI. Strip plots: median and IQR. Each dot represents one implant. *p = 0.024 (Astra Tech Osseospeed vs Nobel TiUnite); *p* = 0.047 (Astra Tech Osseospeed vs Others).

We initially focused our analysis on trace elements in the entire specimens (Fig. 2b (i), Fig. 3a (i), Table 2 (i), Supplementary Fig. 1). Due to the extent of the inflammatory process in peri-implantitis sites, the overall size of the soft tissue specimens was significantly larger in the peri-implantitis group than in the reference site group. Despite these differences in area and volume, no significant differences were observed in terms of number of titanium micro-particles or % area occupied by particles. The assessment of volumetric densities (*n* of particles/mm^3^) revealed similar results for peri-implantitis and reference sites. Thus, the level of inflammation in peri-implant tissues appears not to be linked to the density of titanium micro-particles.

**Table 2:** Titanium micro-particles in different regions of interest (ROI)

	Peri-implantitis		Reference implant		
	*mean*	*sd*	*mean*	*sd*	*p*
Biopsy Thickness (µm)	20.56	4.16	21.11	3.23	
i. Entire Specimen					
ROI Area (mm^2^)	18.72	8.48	8.77	4.19	0.0002
ROI Volume (mm^3^)	0.4	0.24	0.18	0.08	0.0003
Number of particles (*n*)	68	58	37	32	
Area occupied by particles (%)	0.11	0.1	0.15	0.14	
Volumetric density (*n* particles/mm^3^)	190	140	210	150	
ii. Zone 1					
ROI Area (mm^2^)	4.82	2.51	2.99	0.97	0.0006
ROI Volume (mm^3^)	0.1	0.08	0.06	0.02	0.0123
Number of particles (*n*)	27	38	15	18	
Area occupied by particles (%)	0.16	0.18	0.18	0.15	
Volumetric density (*n* particles/mm^3^)	260	308	260	231	
iii. Zone 2					
ROI Area (mm^2^)	5.68	2.06	3.15	1.32	0.0003
ROI Volume (mm^3^)	0.12	0.06	0.07	0.03	0.001
Number of particles (*n*)	19	19	14	15	
Area occupied by particles (%)	0.09	0.09	0.18	0.24	
Volumetric density (*n* particles/mm^3^)	159	125	229	191	
iv. Zone 3^a^					
ROI Area (mm^2^)	8.07	5.71	3.09	2.75	0.0008
ROI Volume (mm^3^)	0.17	0.13	0.06	0.05	0.0008
Number of particles (*n*)	22	23	9	16	
Area occupied by particles (%)	0.1	0.12	0.06	0.1	0.002
Volumetric density (*n* particles/mm^3^)	152	182	116	131	

Wilcoxon signed-rank test.

^a^*n* = 15 in Reference implant sites.

Additional analyses were performed to assess the spatial distribution of titanium elements in tissues (Fig. 2b (ii), Fig. 3a (ii–iv), Table 2 (ii–iv), Supplementary Fig. 1). Using the lateral borderline of the sample that indicated the implant/tissue interface, each specimen was divided into three parallel zones. “Zone 1” extended from the implant/tissue interface up to 1 mm and “zone 2” from 1 to 2 mm. The remaining tissue fraction, “zone 3”, extended from 2 mm and onwards. Due to variation in size, not all reference-site specimens presented with a dimension that extended beyond zone 2. No significant differences in volumetric densities of titanium micro-particles were detected in zones 1 or 2 between peri-implantitis and reference sites. While no significant differences in volumetric densities of titanium micro-particles between specimen groups were observed in zone 3, the percentage area occupied by particles was significantly larger in peri-implantitis sites than in reference implant sites (0.10% ± 0.12 versus 0.06% ± 0.10, respectively). This difference may be related to the varying size of zone 3 between groups.

**Fig. 4:**
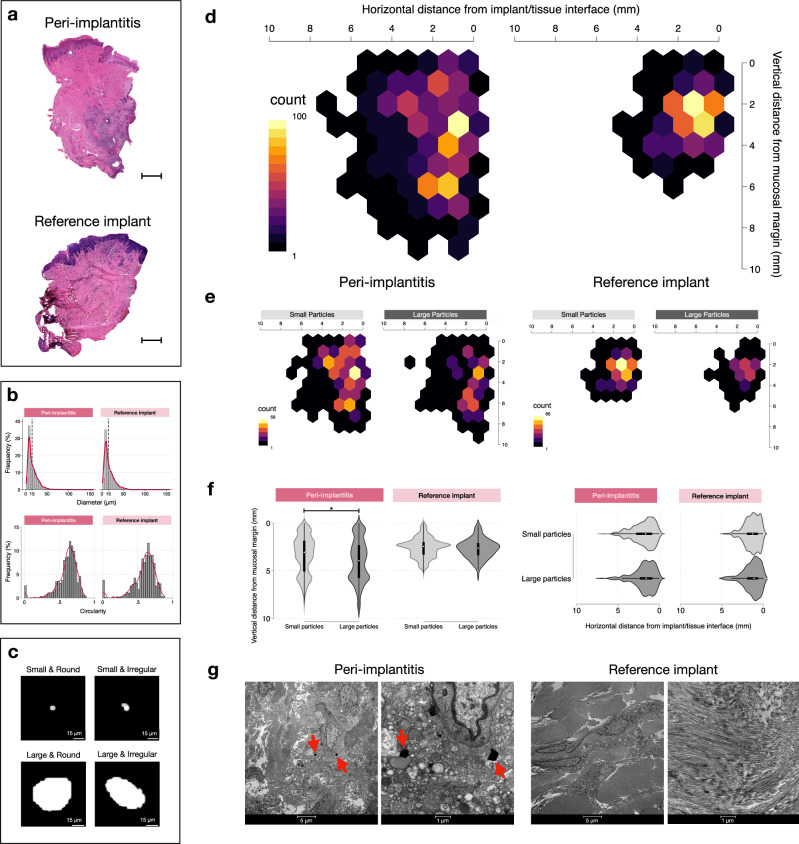
Results of μ-PIXE analysis (continued). **a** Representative Hematoxylin & Eosin micrographs in one peri-implantitis and one reference implant specimen. The implant/tissue interface is found on the right side of the samples. Magnification 20×. Scale bars: 1 mm. **b** Distribution of diameter and circularity values of titanium particles in peri-implantitis (*n* = 1228) and reference implant (*n* = 663) sites. K-densities represented by red continuous lines. **c** Representative images of titanium micro-particles depending on size and shape. **d** Heatmaps illustrating the distribution of titanium micro-particles based on results from linear measurements in peri-implantitis and reference implant specimens. **e** Heatmaps representing the distribution of small and large particles based on results from linear measurements in peri-implantitis and reference implant specimens (peri-implantitis: small particles *n* = 755, large particles *n* = 473; reference implants: small particles *n* = 41, large particles *n* = 251). **f** Violin plots representing the distribution of small and large particles based on results from linear measurements in peri-implantitis and reference implant specimens. *p < 0.0001, Mann-Whitney U test. **g** Representative TEM images illustrating sections from peri-implantitis and reference implant specimens. Red arrows indicate metal-like particles. Magnification: 1600× & 6700×.

In a further analysis we evaluated potential differences between connective tissue compartments occupied by an inflammatory cell infiltrate and connective tissue areas devoid of inflammation (Fig. 2b (iii); Fig. 3a (v–vi), Table 3 (v–vi), Supplementary Fig. 1). Thus, one “zone with inflammation” and one “zone without inflammation” were determined in each specimen by superimposing the titanium maps over images obtained from 5-µm-thick Hematoxylin/Eosin-stained sections (Fig. 4a). The data analysis revealed no significant differences in volumetric densities of Ti-elements between inflamed and non-inflamed connective tissue compartments in peri-implantitis and reference implant sites.

**Table 3:** Titanium micro-particles in different regions of interest (ROI)

	Zone with inflammation		Zone without inflammation		
	*mean*	*sd*	*mean*	*sd*	*p*
v. Peri-implantitis					
ROI Area (mm^2^)	10.99	6.88	5.69	3.49	0.0123
ROI Volume (mm^3^)	0.23	0.18	0.12	0.08	0.0139
Number of particles (*n*)	47	51	17	21	
Area occupied by particles (%)	0.14	0.12	0.11	0.18	
Volumetric density (*n* particles/mm^3^)	222	198	152	189	
vi. Reference implant					
ROI Area (mm^2^)	3.21	1.41	3.99	3.2	
ROI Volume (mm^3^)	0.07	0.03	0.08	0.06	
Number of particles (*n*)	23	26	12	21	
Area occupied by particles (%)	0.22	0.17	0.17	0.39	0.0342
Volumetric density (*n* particles/mm^3^)	344	326	157	217	

Wilcoxon signed-rank test.

To explore the variation in volumetric densities of titanium micro-particles between patients, a linear regression analysis was performed as a final step of evaluation (Fig. 3b, c; Supplementary Table 1). Implant characteristics (implant system, years in function, implant length, implant diameter, jaw, location, tooth-reconstruction type) and findings from the clinical examination prior to biopsy collection (probing pocket depth, marginal bone level) were entered as independent variables. The analysis disclosed that implant system had a significant impact on volumetric densities in the defined sub-area termed “zone 1” (closest zone to the implant/tissue interface) (Fig. 3c).

While titanium dominated in all specimens, iron (Fe) was not considered due to potential contamination from instruments during collection and processing of samples. Very few sections demonstrated isolated alloying elements of Fe, such as Co-Cr. For control purposes, μ-PIXE analysis of gingival tissue specimens obtained from tooth sites with periodontitis in patients without dental implants was performed. No titanium micro-particles were detected in connective tissue portions of these negative control specimens. Occasionally, some few isolated micro-particles (diameter < 15 μm) were observed in the pocket epithelium.

A comprehensive characterization of each single titanium micro-particle was performed. No statistically significant differences in area, diameter, circularity and Feret diameters of particles were observed between peri-implantitis and reference sites (Table 4). In addition, the vertical distance from the mucosal margin and the horizontal distance from the implant/tissue interface was measured for each particle (Fig.2b (iv-v)). Reference lines corresponding to the implant/tissue interface and the mucosal margin were outlined and perpendicular distances between the lines and the micro-particles were automatically traced and measured using the Image-Pro Premier software (IPP, version 10; Media Cybernetics Inc., Rockville, MD, USA). The titanium micro-particles were categorized as small (diameter ≤15 µm) or large (diameter > 15 µm). About 60% of particles in both groups had a diameter of ≤15 µm and a circularity level of ≥0.6 (Fig. 4b). Pictographs of different types of titanium micro-particles of varying sizes and shapes are shown in Fig. 4c. The heatmap in Fig. 4d illustrates a similar horizontal and vertical distribution of particles in samples from peri-implantitis and reference implant sites. There were no differences in the horizontal or vertical distribution of small and large particles across any of the specimen groups (Fig. 4f).

**Table 4:** Characteristics of titanium micro-particles in peri-implantitis (*n* = 1228) and reference implant (*n* = 663) sites

	Peri-implantitis		Reference implant	
	mean	sd	mean	sd
Area (µm2)	359.08	652.84	384.95	981.48
Diameter (µm)	16.17	12.03	16.42	12.77
Circularity [0−1]	0.73	0.16	0.71	0.18
Caliper mean (µm)	18.18	14.1	18.77	15.42
Caliper max (µm)	20.93	16.66	21.84	18.85
Caliper min (µm)	14.07	11.21	14.29	11.49

### Transmission electron microscopy (TEM)

As titanium micro-particles may occur in extra- and intra-cellular compartments, ultra-thin sections were prepared for TEM analysis. Representative micrographs are shown in Fig. 4g. Very few metal-like micro-particles were observed in TEM sections from peri-implantitis sites. All the observed metal-like micro-particles were located in the extra-cellular compartment. Well-oriented collagen fibers and numerous fibroblasts were found in sections from reference sites. Sections from peri-implantitis sites exhibited evident signs of tissue degradation and elevated numbers of inflammatory cells. It should be kept in mind that limitations of the technique, e.g., the ultra-thin dimension of the section thickness of about 700 Å, probably influenced the likelihood for particle detection.

### Differential gene expression

We analyzed differential gene expression in cells residing in the extensive inflammatory process of peri-implantitis specimens to investigate whether the presence of titanium micro-particles influenced the function of inflammatory cells. Based on the assessment of volumetric densities of titanium micro-particles in the “zone with inflammation”, we identified two distinct groups of specimens. The group presenting with higher volumetric densities than the mean value (190 particles/mm^3^) was termed “Ti-high”, while the group with lower densities than the mean value was termed “Ti-low”.

**Fig. 5:**
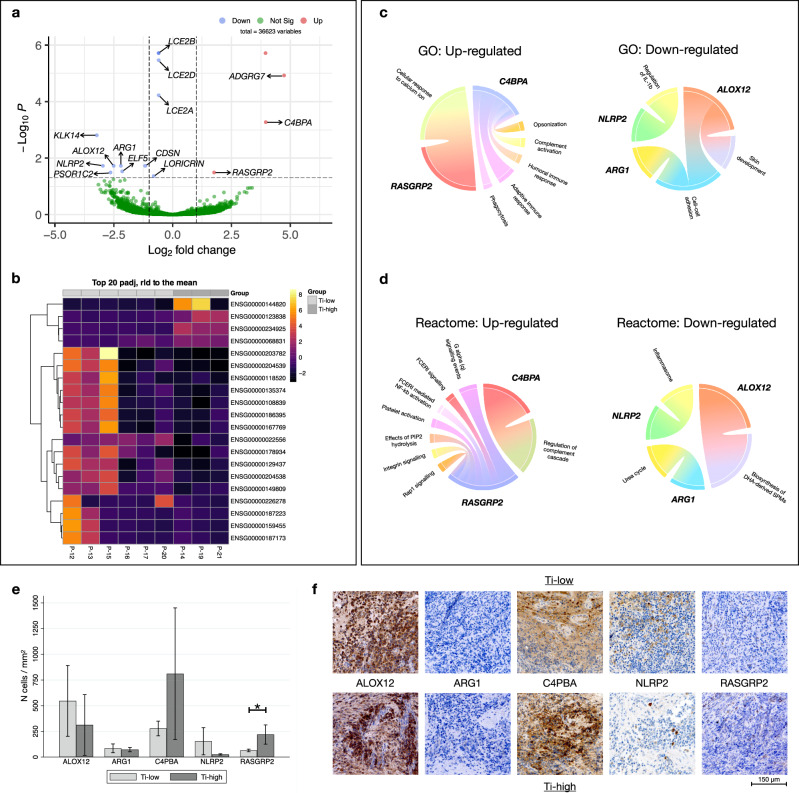
Results of RNA-sequencing and immunohistochemical analyses. **a** Volcano plot representing DEGs found comparing Ti-high and Ti-low. Up-regulated genes (red), down-regulated genes (blue). **b** Heatmap illustrating the 20 genes with strongest gene expression levels (Log_2_FC) found comparing Ti-high and Ti-low. **c** Circo-plots illustrating up- and down-regulated Gene Ontology (GO) pathways and relative genes (in bold). **d** Circo-plots illustrating up- and down-regulated Reactome pathways and relative genes (in bold). **e** Bar graphs illustrating the density of positive cells in Ti-low (*n* = 6) and Ti-high (*n* = 3) groups. Mean and 95% CI. *p = 0.0253, Mann-Whitney U test. **f** Representative pictographs of positive cells in Ti-low and Ti-high specimens. Magnification × 400.

Among a total of 36623 entries in the comparison between the Ti-high and Ti-low groups, we identified 16 differentially expressed genes (DEGs). One up-regulated (*ENSG00000226278*) and one down-regulated entry (*ENSG00000234925*) were identified as putative genes without any specific geneID. The volcano plot in Fig. 5a illustrates 3 significantly up-regulated genes (*ADGRG7, C4BPA, RASGRP2*) and 11 down-regulated genes (*LCE2B, LCE2D, LCE2A, CDSN, LORICRIN, ARG1, NLRP2, ALOX12, KLK14, PSORS1C2, ELF5*). DEGs with corresponding Ensembl symbols presenting with the highest gene-expression levels (Log_2_ fold change) are shown in the heatmap in Fig. 5b.

Gene ontology (GO) and Reactome enrichment analyses were performed to explore their biological functions (Fig. 5c, d). DEGs were enriched in 10 GO biological pathways and functions related to the regulation of the immune response and skin development were the most abundant. The significant biological pathways based on the Reactome analysis revealed significant differences between the Ti-high and Ti-low groups, particularly in regard to “regulation of complement cascade”, “platelet activation and signaling” and “keratinization”. Interestingly, gene *RASGRP2*, which was up-regulated in the Ti-high group, was found to be part of the FCERI signaling and NF-kb activation pathways.

### Immunohistochemical detection of cell markers

For validation of gene expression findings in peri-implantitis lesions, we performed immunohistochemical analyses (IHC) to detect cells exhibiting specific markers (Fig. 5f). The selected markers corresponded to the 5 most conspicuous up- and down-regulated genes observed in the RNA-sequencing analysis. Using the IPP software the densities of positive cells within the ICT were determined (*n* cells/mm^2^). The analysis revealed that in peri-implantitis samples, the Ti-high group presented with significantly higher cellular densities of RASGRP2-positive cells than the Ti-low group (Fig. 5e). Although not statistically significant, the density of C4PBA-positive cells was about 3 times higher in Ti-high than in Ti-low groups of specimens (*p* = 0.0526). No significant differences in cellular densities were observed for the other markers between the two groups.

## Discussion

The present study demonstrated that titanium micro-particles were consistent findings in soft tissues surrounding dental implants and that their occurrence was unrelated to the health status of the peri-implant tissues. By adopting a study design that utilized paired samples, each derived from the very same patient, inter-subject variability was of limited concern. In addition, the in-depth localization, quantification, and characterization of titanium micro-particles within peri-implant tissues was feasible through the deployment of an advanced and reliable methodology. While μ-PIXE allows the analysis of metal particles within biological tissues owing to its inherent capacity to reveal elemental compositions with high precision at the micron-level^35^, other techniques (e.g., synchrotron radiation) may provide greater spatial resolution or penetration depth and allow analysis of particles down to the nano-size scale^36^. The focus on titanium micro-particles in peri-implant tissues in the present study was based on the concept that 88–99.5% nominal weight of the dental implant composition is made of titanium, although different alloys are commonly used, e.g., Ti-6Al-4V and Ti-6Al-7Nb^37^.

We analyzed 4–6 mm wide tissue samples and demonstrated that the majority of titanium micro-particles were located in a 2-mm wide tissue compartment close to the implant/tissue interface. Beyond this 2-mm wide zone, towards the deeper portions of the tissue sample, a gradient decrease in density of titanium micro-particles was observed. In addition, there were no evident differences in the morphology of the titanium micro-particles between peri-implantitis and reference sites. Most micro-particles displayed a circular-like appearance with contained diameters.

The current analysis was based on pairs of tissue samples obtained from 18 patients with peri-implantitis. Limitations in terms of sample size and inclusion criteria resulted in an uneven distribution regarding implant systems among the study sample. Nevertheless, our data suggest that the type of implants influenced the density of titanium micro-particles within the tissue samples. This observation, however, must be interpreted with care as implant systems differ in several aspects. Differences include not only implant geometry and surface characteristics but also the surgical protocol, by which the devices are to be installed in the bone. Thus, an undersized preparation of the implant bed during osteotomy may result in an increased friction between the bony wall and the screw-shaped implant during installation. Micro-particles may thereby be dislodged and subsequently become encapsulated in the supra-crestal connective tissue. The observation that time in function of the implant device did not influence the concentration of titanium micro-particles in peri-implant tissues in the present study, supports the concept that the deposition of micro-particles is probably a result of early events. It should be pointed out, however, that analysis of human tissue samples presents with obvious limitations in detecting pathways for the accumulation of micro-particles. On the other hand, data reported in an in vitro experiment on the wear of metal particles from commercially available dental implants during installation in artificial bone, however, corroborate the notion on friction^38^. X-ray-fluorescence spectroscopy analysis revealed metal debris detached from the implant body with a greater deposition around the coronal than around the apical parts of the devices.

The question whether the presence of titanium micro-particles in peri-implant tissues influences the local host response to infection was investigated by comparing RNA-seq data of peri-implantitis specimens with different volumetric densities of micro-particles. Only minor differences in gene expression were observed between tissue samples containing high or low densities of micro-particles. This was evidenced by the observation of only 3 up-regulated and 11 down-regulated genes out of >36000 examined entries. The 14 genes were all associated with pathways specific for inflammation. As a validation procedure, immunohistochemistry analysis was made on 5 markers selected from the 2 most up- regulated and 3 most down-regulated genes. RNA-seq and IHC data showed consistency concerning up- and down-regulated genes. In addition, proportions of RASGRP2-positive cells were significantly larger in tissue samples with high densities of titanium particles than those with low densities. Members of the RAS family are signaling proteins involved in several cellular processes, such as cell growth, differentiation and proliferation^39^. In addition, they are recognized as important biomarkers regulating the chemotaxis and adhesion of leukocytes as well as the activation of platelets and endothelial cells^40,41^. Notably, the RNA-seq analysis and IHC validation in the present study were performed on a limited number of samples collected purely from diseased sites. Thus, inter-individual variability in inflammatory conditions must be considered. Taken together, the results from the gene expression and immunohistochemical analyses appear to reflect a complex and severe inflammatory state of peri-implantitis specimens.

In conclusion, titanium micro-particles appear not to be a characteristic specific for peri-implantitis but rather a common occurrence in peri-implant tissues in patients with dental implants. While associations between high densities of titanium micro-particles and the local host response appear to be restricted to few pro-inflammatory mediators, it needs to be kept in mind that tissue samples from sites with and without peri-implantitis in the same individual presented with similar densities of titanium micro-particles.

## Supplementary information

Supplementary Information

Description of Additional Supplementary Files

Supplementary Data

Reporting summary

## Data Availability

Bulk RNA-seq data were deposited at the European Genome-Phenome Archive (https://ega-archive.org/studies/EGAS50000000369 and https://ega-archive.org/datasets/EGAD50000000544). Raw datasets for the μ-PIXE and IHC analyses are provided as a “Supplementary Data” file.
