# Supplementary material for: Titanium micro-particles are commonly found in soft tissues surrounding dental implants
Source: Commun Med (Lond). 2025 Mar 18;5:78. doi: 10.1038/s43856-025-00756-3 (PMC11920262; doi:10.1038/s43856-025-00756-3)
Supplement: Supplementary file 1 — Supplementary Information [file 43856_2025_756_MOESM1_ESM.pdf]

Supplementary table 1. Results from linear regression analysis.

ROI	Variables	Coefficient	*p*	[95% CI]	
**Entire Specimen**					
	**Continuous variables**				
	Probing pocket depth	-7.13	0.453	-26.71	12.46
	Radiographic bone level	-9.28	0.328	-28.71	10.15
	Implant years in function	-5.94	0.13	-13.82	1.94
	Implant diameter	13.86	0.781	-89.64	117.37
	Implant length	-3.83	0.698	-24.38	16.73
	**Categorical variables**				
	Jaw				
	Mandible	(base)			
	Maxilla	-63.99	0.222	-170.55	42.58
	Implant position				
	Anterior	(base)			
	Posterior	58.54	0.275	-51.06	168.14
	Tooth reconstruction type				
	Single crown	(base)			
	Two-unit bridge	-102.4	0.299	-304.09	99.28
	Three-unit bridge	-190.19	0.057	-386.79	6.42
	Multiple-unit bridge	-36.98	0.793	-329.95	255.99
	Full-arch bridge	-45.45	0.705	-294.61	203.7
	Overdenture	-92.96	0.424	-322.22	146.29
**Zone 1**					
	Implant system				
	Astra Osseospeed	(base)			
	Nobel TiUnite	237.26	**0.024**	34.89	439.63
	Others	236.3	**0.047**	2.93	469.68

Supplementary table 2. Primary antibodies used in the immunohistochemical analysis.

Antibody	Origin	Type	Isotype	Dilution	Company
ALOX12	Rabbit	Polyclonal	aa618-650	1:75	LS-Bio
ARG1	Rabbit	Polyclonal	IgG	1:100	Invitrogen
C4BPA	Rabbit	Polyclonal	aa470-499	1:150	LS-Bio
NLRP2	Rabbit	Polyclonal	IgG	1:35	LS-Bio
RASGRP2	Rabbit	Polyclonal	IgG	1:250	GeneTex

Supplementary Fig. 1.

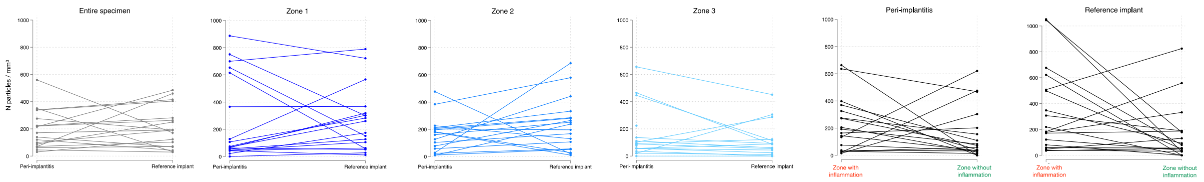

Strip plots illustrating volumetric densities of Ti micro-particles in the different ROIs in peri-implantitis and reference implant sites. Each dot represents one implant and paired implants (from the same patient) are connected by straight lines.

Supplementary Fig. 2.

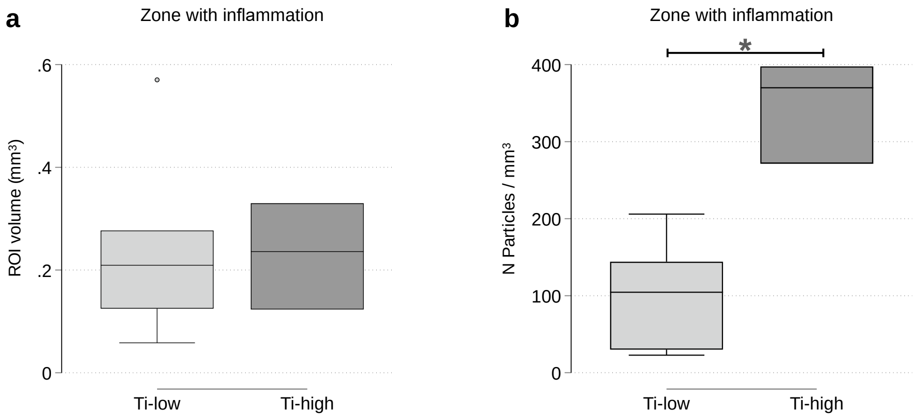

***a)** Boxplots illustrating the volume (mm^3) of the "zone with inflammation" ROI in Ti-low and Ti-high groups. Median and IQR. Circles represent outliers.*
***b)** Boxplots illustrating the volumetric density of titanium micro-particles in the "zone with inflammation" ROI in Ti-low and Ti-high groups. Median and IQR. *$p<0.05$, Mann-Whitney U test*
