# Supplementary material for: Titanium micro-particles are commonly found in soft tissues surrounding dental implants
Source: Commun Med (Lond). 2025 Mar 18;5:78. doi: 10.1038/s43856-025-00756-3 (PMC11920262; doi:10.1038/s43856-025-00756-3)
Supplement: Supplementary file 2 — Description of Additional Supplementary Files [file 43856_2025_756_MOESM2_ESM.pdf]

Description of Additional Supplementary Files

File name: Supplementary Data

File description: Raw datasets for the μ-PIXE and IHC analyses
