# Supplementary material for: Titanium micro-particles are commonly found in soft tissues surrounding dental implants
Source: Commun Med (Lond). 2025 Mar 18;5:78. doi: 10.1038/s43856-025-00756-3 (PMC11920262; doi:10.1038/s43856-025-00756-3)
Supplement: Supplementary file 4 — Reporting summary [file 43856_2025_756_MOESM4_ESM.pdf]

nature portfolio

Corresponding author(s): Tord Berglundh

Last updated by author(s): Feb 1, 2024

Reporting Summary

Nature Portfolio wishes to improve the reproducibility of the work that we publish. This form provides structure for consistency and transparency in reporting. For further information on Nature Portfolio policies, see our Editorial Policies and the Editorial Policy Checklist.

Statistics

For all statistical analyses, confirm that the following items are present in the figure legend, table legend, main text, or Methods section.

n/a	Confirmed	
☐	☒	The exact sample size (*n*) for each experimental group/condition, given as a discrete number and unit of measurement
☐	☒	A statement on whether measurements were taken from distinct samples or whether the same sample was measured repeatedly
☐	☒	The statistical test(s) used AND whether they are one- or two-sided<br>*Only common tests should be described solely by name; describe more complex techniques in the Methods section.*
☐	☒	A description of all covariates tested
☐	☒	A description of any assumptions or corrections, such as tests of normality and adjustment for multiple comparisons
☐	☒	A full description of the statistical parameters including central tendency (e.g. means) or other basic estimates (e.g. regression coefficient) AND variation (e.g. standard deviation) or associated estimates of uncertainty (e.g. confidence intervals)
☐	☒	For null hypothesis testing, the test statistic (e.g. *F*, *t*, *r*) with confidence intervals, effect sizes, degrees of freedom and *P* value noted<br>*Give P values as exact values whenever suitable.*
☒	☐	For Bayesian analysis, information on the choice of priors and Markov chain Monte Carlo settings
☐	☒	For hierarchical and complex designs, identification of the appropriate level for tests and full reporting of outcomes
☒	☐	Estimates of effect sizes (e.g. Cohen's *d*, Pearson's *r*), indicating how they were calculated

Our web collection on statistics for biologists contains articles on many of the points above.

Software and code

Policy information about availability of computer code

Data collection

For micro-PIXE: GeoPIXE software (version 8.6, CSIRO, Australia)
For IHC: Image-Pro Premier (IPP, version 10, Media Cybernetics Inc., Rockville, MD, USA).
For RNA seq: NovaSeq 6000 (Illumina).
For clinical measurements: Microsoft Excel (206, ver 16.16.27), FileMaker ()

Data analysis

micro-PIXE:
Image-Pro Premier (IPP, version 10, Media Cybernetics Inc., Rockville, MD, USA).
Microsoft Excel (206, ver 16.16.27),
Stata

IHC:
Image-Pro Premier (IPP, version 10, Media Cybernetics Inc., Rockville, MD, USA).
Microsoft Excel 206, ver 16.16.27
Stata

RNA seq:
The quality of the reads was examined using fastqc/0.11.9 (Andrews S., 2010), the resulting quality reports were summarized using MultiQC/1.9 (Etwels et al., 2016). The reads were quality filtered using Trim Galore/0.4.0 (Krueger et al., 2021) and adapters were removed using Cutadapt/1.9 (Martin M., 2011). The quality filtered reads were aligned towards the human reference genome GRCh38.109 using STAR/2.7.10b (Dobin et al., 2012). Infer experiment within RSeQC/5.0.1 (Wang et al., 2012) was used to extract the strandness of the data. Featurecounts within the subread/2.0.4 package (Liao et al., 2014) was used to gather the gene counts. The differential expression analysis

was run in the R/4.1.3 package (R Core Team, 2022) DESeq2/1.34.0 (Love et al., 2014). The package pheatmap/1.0.12 (Kolde et al., 2019) was used to generate the heatmaps. ClusterProfiler/4.2.2 (Wu et al., 2021) was used to perform the overrepresentation analysis for Gene Ontology (http:// geneontology.org; Ashburner et al., 2011) and Reactome (https://reactome.org; Jassal et al., 2020)

For manuscripts utilizing custom algorithms or software that are central to the research but not yet described in published literature, software must be made available to editors and reviewers. We strongly encourage code deposition in a community repository (e.g. GitHub). See the Nature Portfolio guidelines for submitting code & software for further information.

Data

Policy information about availability of data

All manuscripts must include a data availability statement. This statement should provide the following information, where applicable:

- Accession codes, unique identifiers, or web links for publicly available datasets
- A description of any restrictions on data availability
- For clinical datasets or third party data, please ensure that the statement adheres to our policy

Bulk RNA-seq data were deposited at the European Genome-Phenome Archive (https://ega-archive.org/studies/EGAS50000000369 and https://ega-archive.org/datasets/EGAD50000000544). All other data that support the findings of this study are available from the corresponding author upon reasonable request.

Research involving human participants, their data, or biological material

Policy information about studies with human participants or human data. See also policy information about sex, gender (identity/presentation), and sexual orientation and race, ethnicity and racism.

Reporting on sex and gender	Both men and women were recruited in the study. Only 'biological sex' was recorded for the purpose of the present study.
Reporting on race, ethnicity, or other socially relevant groupings	Ethnicity data were not recorded for the purpose of the present study.
Population characteristics	All patient-related characteristics are reported in details in Table 1.<br>In brief, 21 patients with dental implant-supported crowns or prostheses [17 women/3 men; mean age 72 years old (SD 11 years; range: 45-90 years), 17 non-smokers/ 3 current smokers, 2 patients with diabetes type 2] were consecutively recruited from the Specialist Clinic of Periodontics in Gothenburg, Public Dental Services, Region Västra Götaland, Sweden. Before enrolment, all subjects received detailed information about the study protocol and signed an informed consent.
Recruitment	This was a cross-sectional observational study. Patients recruitment was based on specific inclusion/exclusion criteria.<br><br>Each patient had to present with ≥1 dental implants exhibiting (i) severe inflammation together with evident destruction of supporting bone (peri-implantitis) and (ii) ≥1 adjacent implants with clinically healthy conditions or mild inflammation without evident bone loss (reference sites). The clinical characteristics for peri-implantitis sites included peri-implant probing pocket depth (PPD) of ≥7 mm, bleeding and/or suppuration on probing (BoP/SoP) and radiographically assessed bone levels of ≥3 mm. Reference implant sites showed either presence or absence of BoP and PPD ≤5 mm.
Ethics oversight	The study protocol was approved by the Swedish Ethical Review Authority (Dnr 2021-00508).

Note that full information on the approval of the study protocol must also be provided in the manuscript.

Field-specific reporting

Please select the one below that is the best fit for your research. If you are not sure, read the appropriate sections before making your selection.

☒ Life sciences ☐ Behavioural & social sciences ☐ Ecological, evolutionary & environmental sciences

For a reference copy of the document with all sections, see nature.com/documents/nr-reporting-summary-flat.pdf

Life sciences study design

All studies must disclose on these points even when the disclosure is negative.

Sample size	No sample size calculation was performed due to lack of information on densities of metal particles in reference sites from previous publications.The strategy of using paired specimens (1 from a diseased and 1 from adjacent reference site, each obtained from the very same patient) served the purpose of reducing biological and technical variability among samples.
Data exclusions	Three pairs of soft tissue samples deemed for micro-PIXE analysis (n=6) were excluded due to complications during sample preparation.<br><br>Pre-determined Quality Control criteria for RNA-sequencing were applied via the Tapestation 4200 system (Agilent, USA). Quality checks revealed that all samples presented with optimized concentrations of total RNA [36-463 ng/uL] and RIN scores [5-9].<br>Libraries were sequenced with an avarage depth of 59.3 (+/- 11.4) Mreads/sample. The quality of the reads was examined using FastQC (ver.

	0.11.9) and the resulting quality reports summarized via MultiQC (ver. 1.9). The reads were quality filtered with Trim Galore (ver. 0.4.0) and adapters were removed with Cutadapt (ver. 1.9).
Replication	Harvesting of soft tissue biopsies was not replicated due ethical considerations.<br>We present initial analysis to illustrate the utility of micro-PIXE combined with IHC and RNA-seq analyses.
Randomization	No randomization procedures were involved. This was a cross-sectional observational study.
Blinding	All samples were assigned a unique study ID (the key held in a locked safe at the Specialist Clinic of Periodontics in Gothenburg, Public Dental Services, Region Västra Götaland, Sweden, only accessible to authorized personnel). All examiners involved in the micro-PIXE, TEM, IHC, RNA-seq analyses were blinded to the any patient information.

Reporting for specific materials, systems and methods

We require information from authors about some types of materials, experimental systems and methods used in many studies. Here, indicate whether each material, system or method listed is relevant to your study. If you are not sure if a list item applies to your research, read the appropriate section before selecting a response.

Materials & experimental systems

n/a	Involved in the study
☐	☒ Antibodies
☒	☐ Eukaryotic cell lines
☒	☐ Palaeontology and archaeology
☒	☐ Animals and other organisms
☐	☒ Clinical data
☐	☒ Dual use research of concern
☒	☐ Plants

Methods

n/a	Involved in the study
☒	☐ ChIP-seq
☒	☐ Flow cytometry
☒	☐ MRI-based neuroimaging

Antibodies

Antibodies used	Anti-ALOX12 (LS-Bio), anti-ARG1 (Invitrogen), anti-C4BPA (LS-Bio), anti-NLRP2 (LS-Bio), anti-RASGRP2 (GeneTex).<br>Details are provided in Supplementary table 2.
Validation	All antibodies were commercially available, validated and were used in previous studies, as per the references available in manufacturer's websites.

Clinical data

Policy information about clinical studies

All manuscripts should comply with the ICMJE guidelines for publication of clinical research and a completed CONSORT checklist must be included with all submissions.

Clinical trial registration	This was not a clinical trial.
Study protocol	The study protocol was approved by the Swedish Ethical Review Autority (Dnr 2021-00508). Full methodological details are provided in the manuscript.
Data collection	A total of 21 patients were recruited in the study. Recruitement took place between May 2021 and June 2022.
Outcomes	Primary outcome: volumetric density of titanium micro-particles (n particles/mm3) in soft tissue biopsies harvested at dental implants sites with and without peri-implantitis. Method: micro-PIXE analysis and ImagePro Premiere analysis.<br><br>Secondary outcomes:<br>- Localization and characterization of titanium micro-partciles within different regions of interes. Method: micro-PIXE analysis, ImagePro Premiere analysis, TEM analysis.<br>- Influence of clinical- and implant-related characteristics on volumetric densities of titanium micro-particles. Method: linear regression analysis .<br>- Differentially expressed genes between peri-implantitis sites presenting with high or low densities of titanium micro-particles. Method: RNA-sequencing and IHC.

Dual use research of concern

Policy information about dual use research of concern

Hazards

Could the accidental, deliberate or reckless misuse of agents or technologies generated in the work, or the application of information presented in the manuscript, pose a threat to:

No	Yes	
☒	☐	Public health
☒	☐	National security
☒	☐	Crops and/or livestock
☒	☐	Ecosystems
☒	☐	Any other significant area

Experiments of concern

Does the work involve any of these experiments of concern:

No	Yes	
☒	☐	Demonstrate how to render a vaccine ineffective
☒	☐	Confer resistance to therapeutically useful antibiotics or antiviral agents
☒	☐	Enhance the virulence of a pathogen or render a nonpathogen virulent
☒	☐	Increase transmissibility of a pathogen
☒	☐	Alter the host range of a pathogen
☒	☐	Enable evasion of diagnostic/detection modalities
☒	☐	Enable the weaponization of a biological agent or toxin
☒	☐	Any other potentially harmful combination of experiments and agents

Plants

Seed stocks	*Report on the source of all seed stocks or other plant material used. If applicable, state the seed stock centre and catalogue number. If plant specimens were collected from the field, describe the collection location, date and sampling procedures.*
Novel plant genotypes	*Describe the methods by which all novel plant genotypes were produced. This includes those generated by transgenic approaches, gene editing, chemical/radiation-based mutagenesis and hybridization. For transgenic lines, describe the transformation method, the number of independent lines analyzed and the generation upon which experiments were performed. For gene-edited lines, describe the editor used, the endogenous sequence targeted for editing, the targeting guide RNA sequence (if applicable) and how the editor was applied.*
Authentication	*Describe any authentication procedures for each seed stock used or novel genotype generated. Describe any experiments used to assess the effect of a mutation and, where applicable, how potential secondary effects (e.g. second site T-DNA insertions, mosiacism, off-target gene editing) were examined.*
